# Internet-Based Sharing Nurse Program and Nurses’ Perceptions in China: Cross-Sectional Survey

**DOI:** 10.2196/16644

**Published:** 2020-07-22

**Authors:** Rendong Huang, Mei Xu, Xiuting Li, Yinping Wang, Bin Wang, Naixue Cui

**Affiliations:** 1 School of Nursing Cheeloo College of Medicine Shandong University Jinan China; 2 Jinan Shizhong People’s Hospital Jinan China; 3 Jinan Central Hospital Jinan China; 4 Peking University Health Science Center Beijing China

**Keywords:** sharing nurse, home visiting, internet plus nursing program, perception, China

## Abstract

**Background:**

China is currently piloting a “Sharing Nurse” program that aims to increase the accessibility of nursing services to at-home patients by enabling patients to order nursing services using mobile apps or online platforms.

**Objective:**

This study aims to assess nurses’ perceptions of the Sharing Nurse program, including their acceptance, concerns, needs, and willingness to take part in the program.

**Methods:**

A total of 694 nurses participated in the questionnaire survey. The survey collected their sociodemographic and work-related information and their perceptions of the Sharing Nurse program using a self-developed questionnaire.

**Results:**

The 694 respondents agreed that the Sharing Nurse program could provide patients with better access to nursing care (n=483, 69.6%). Their main concerns about the program were unclear liability division when medical disputes occur (n=637, 90.3%) and potential personal safety issues (n=604, 87%). They reported that insurance (n=611, 88%), permits from their affiliated hospital (n=562, 81.0%), clear instructions concerning rights and duties (n=580, 83.6%), real time positioning while delivering the service (n=567, 81.7%), and one-key alarm equipment (n=590, 85.0%) were necessary for better implementation of the program. More than half of the respondents (n=416, 60%) had an optimistic attitude toward the development of the Sharing Nurse program in China. However, only 19.4% (n=135) of the respondents expressed their willingness to be a “shared nurse.” Further analyses found that nurses with a master’s degree or above (χ^2^_3_=28.835, *P*<.001) or from tertiary hospitals (χ^2^_3_=18.669, *P*<.001) were more likely to be aware of the Sharing Nurse program and that male nurses were more willing to be shared nurses (*Z*=–2.275, *P*=.02).

**Conclusions:**

The Chinese Sharing Nurse program is still in its infancy and many refinements are needed before it can be implemented nationwide. Generally, Chinese nurses are positive about the Sharing Nurse program and are willing to participate if the program is thoroughly regulated and supervised.

## Introduction

Home visiting is a strategy to deliver various nursing or medical services to families or individuals within their home environment for an extended period of time [1,2]. Nurse home visits are employed in a number of settings, from managing acute or chronic illnesses to aiding the overall health and well-being of the patient. Home visits are also used to ease adults into outpatient settings from inpatient settings. Home visits play an important role in enhancing health and independency, as well as providing high quality end-of-life care to older adults [3,4].

Home visiting nurses, in the provision of their services, have the opportunity to tailor the services to meet a family’s unique needs more effectively [5,6]. Home visiting programs also allow qualified professionals to build a rapport with families, which may not be possible during other types of interventions [7,8]. Home visiting has also been shown to lead to many positive health outcomes. These include improved daily functioning among adults who are disabled, which may reduce Medicaid medical costs [9]; increase quality of life and increased number of days free of asthma symptoms for patients with asthma [10]; reduce child abuse and neglect; and improve child development and parenting outcomes [11-14].

Home visiting has been widely practiced in the United Kingdom, the United States, and many other developed countries. In the United Kingdom, home visiting programs have been expanded since 2005, delivering services to 16,000 disadvantaged new parents each year [15]. In the United States, about US $1.5 billion had been proposed by President Barack Obama’s 2014 Budget to maintain and expand evidence-based voluntary home visiting services [16]. In Germany, since 2012, the federal and local governments have spent €102 million (US $115 million) each year to expand home visiting programs [17].

Nurse home visiting services in China are generally provided by community health care nurses, which began in the late 1990s [18]. Community health care involves a wide range of services such as disease prevention, rehabilitation, health education, and family planning guidance [19]. However, it is seen that community health care nurses remain at community health centers or stations, waiting passively for patients to arrive [18], instead of going into homes to provide health care, even though home visiting is one of their responsibilities. As a result, the traditional community health care service cannot meet the rising needs of at-home-services propelled by the large and continuous increase in the ageing population [20]. In response to this situation, the online nursing service platform (or “Sharing Nurse” platform)—where patients can request registered nurses to visit them at home and provide nursing services—emerged in China.

The Sharing Nurse platform originated as early as 2016 in the Shandong Province and has since spread to other provinces as well as first-tier cities such as Beijing, Shanghai, Guangzhou, and Shenzhen [21]. However, it did not receive official recognition until 2019. On February 12, 2019, the National Health Commission announced that they were launching a pilot Sharing Nurse program as part of the “Internet Plus” initiative for nursing care in six provinces and metropolitan cities (ie, Beijing, Tianjin, Shanghai, Jiangsu, Zhejiang, and Guangdong), as well as some capital cities and regions across the country [22].

The Sharing Nurse Program refers to the use of internet technology (ie, mobile apps, online websites, or other internet-based platforms) to facilitate home visiting nursing service delivery. To our knowledge, there are currently more than 20 Sharing Nurse apps in China. These apps or websites collect and verify the information of registered nurses who want to be part of the program (“shared nurses”). Clients register on the apps or websites to make an online appointment to reserve or order medical services, choose nurses to deliver services based on their preferences, and decide when and where the services are expected to be delivered. The appointed nurse then arrives at the scheduled time with the required medication and equipment to provide the ordered services [23]. After the service is completed, clients can evaluate the nurse’s service and provide feedback on the platform. Currently, services provided by shared nurses include intramuscular injection, intravenous injection, collecting blood samples, and special care like newborn examinations or urethral catheterization, among others [24]. Unlike the home visiting nurses in many developed countries who are employed by agencies specializing in home visiting services, the Chinese shared nurses, in addition to delivering home visiting services through the sharing nurses platforms, are registered nurses affiliated with approved medical institutions (hospitals, community health care centers, and private clinics), and routinely serve in the medical institutions in which they are employed.

The Sharing Nurse program enables clients to actively use health care services without going to the community care centers or stations. As a new health care delivery mode, the program’s quality and effectiveness is heavily dependent on both nurses and clients. Nurses’ perceptions, such as acceptance of the program, as well as concerns, needs, and perceived benefits of being a shared nurse can greatly influence their willingness and motivation to participate in the program and to provide high-quality care. Eventually the success of the program also depends on how the government addresses the nurses’ concerns and needs. However, research regarding nurses’ perceptions (ie, acceptance, concerns, needs, and perceived benefits) of the Sharing Nurse program is still scarce. To address this gap, we conducted a questionnaire survey to assess nurses’ perceptions of the program, as well as their willingness to be a shared nurse.

## Methods

### Design and Participants

**Figure 1:**
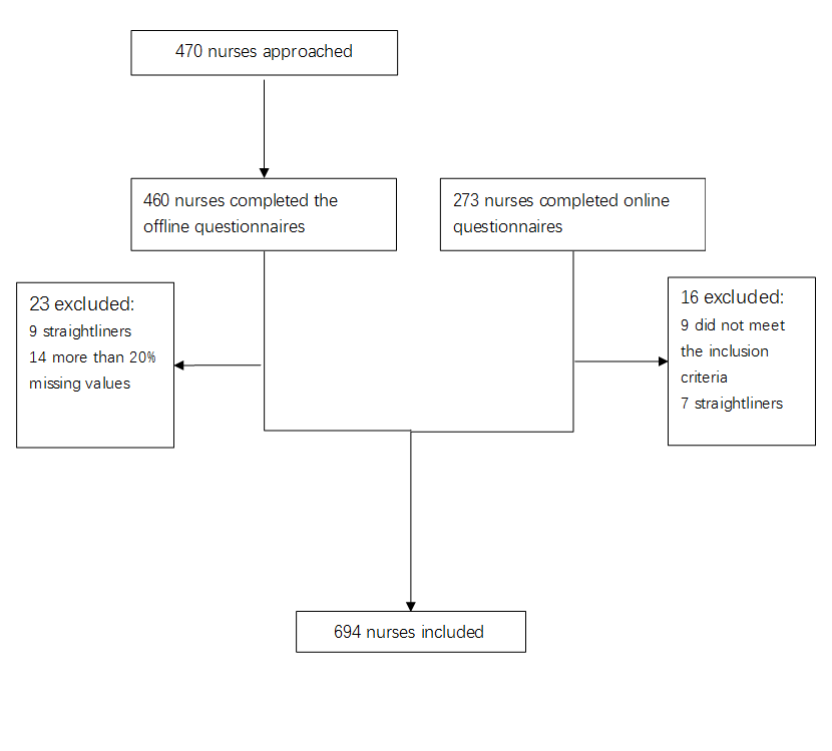
Flow diagram of the participants recruitment and questionnaires screening.

A cross-sectional survey was conducted, both online and offline, in Jinan, China, from March to May 2019. A convenience sampling method was used to recruit registered nurses from two tertiary hospitals, one secondary hospital, seven community health centers, and four private hospitals in the capital city of Shandong Province, where the Sharing Nurse platform originated. The eligibility criterion for the nurses’ participation in this study was more than 1 year of bedside nursing experience. A final sample of 694 nurses completed the questionnaires. The method of inducting participants is illustrated in the flow chart (Figure 1).

### Measurements

#### Sociodemographic Information

The sociodemographic items were age, gender, professional title (nurse, senior nurse, nurse-in-charge, associate director, or director of nurses), years of work experience, educational background, and level of the affiliated hospital (tertiary hospital, secondary hospital, community hospital, or private hospital).

#### Nurses’ Perceptions of the Sharing Nurse Program

We developed a questionnaire to assess nurses’ perceptions of the Sharing Nurse program following these steps. First, we created an initial version of the questionnaire by developing items based on existing literature and interviews with a panel of 20 clinical nurses. Second, an expert panel comprising of two health care researchers, a senior nurse, and a community nurse refined these items. Third, five nurses took a pretest of the questionnaire and modifications were made according to their feedback to obtain the final version of the questionnaire.

The final version of the questionnaire contained five questions concerning nurses’ familiarity with the Sharing Nurse program, whether they have ever been a shared nurse before, and reasons why they do not try to become a “shared nurse”; seven questions about their concerns about the program, rated on a 5-point Likert scale (1=“strongly unconcerned” to 5=“strongly concerned”); seven questions about the possible needs of shared nurses, rated on a 5-point Likert scale (1=“strongly disagree” to 5=“strongly agree”); four questions about the perceived benefits of the program, rated on a 5-point Likert scale (1=“strongly disagree” to 5=“strongly agree”), and one question about their willingness to be a shared nurse (response options including “willing to,” “not willing to until regulations are refined,” and “not willing to even though regulations refined”).

### Data Collection

First, hospitals of different levels were selected to enable us to explore possible variations in nurses’ perceptions of the program across all hospital levels. At each chosen hospital, researchers distributed the questionnaires to nurses on site. To reach out to more nurses, such as those who were not on duty, we also distributed electronic questionnaires (e-questionnaires) in the WeChat work group of the hospitals by using an online questionnaire platform Wenjuanxing. In the e-questionnaire, we added an extra question: “Have you ever attempted this questionnaire before?” at the end of the questionnaire to filter duplicate respondents. The willingness to participate, and thus provide informed consent, was determined at the receipt of the completed questionnaire. This study was approved by the Ethical Committees of Shandong University School of Nursing (Reference Numbers: 2019-R-009).

### Data Analysis

Descriptive statistics were used to describe the respondents’ sociodemographic and work-related information, as well as their responses to the questions regarding their perceptions of the Sharing Nurse program. A comparison analysis was conducted to test whether the perception differed by age, gender, educational background, and hospital levels using a chi-square test, Kruskal-Wallis tests, or the Mann-Whitney U test. For all tests, the significance level was set at *P*≤.05. Data analysis was performed using SPSS 22.0 software (IBM Corp).

## Results

### Demographic and Work-Related Characteristics of the Participants

Of the 694 participants, 672 were female and 22 were male. The age of the participants ranged from 20 to 55 (mean 30.5, SD 6.8) years. The participants had been practicing clinical nursing for an average of 8.5 (SD 7.1) years. The highest percentage of the total 694 nurses (n=313, 45.1%) were from tertiary hospitals, 207 (29.9%) were from secondary hospitals, 81 (11.7%) were from community health care centers, and 114 (16.4%) were from private hospitals. Regarding their educational background, 210 (30.2%) held an associate’s degree, 449 (64.7%) held a bachelor’s degree, and 13 (1.9%) held a masters’ degree or above. Concerning the distribution of nurses according to their different titles, 260 (37.5%) were nurses (junior title for the nursing profession), 275 (39.6%) were senior nurses, 143 (20.6%) were nurses-in-charge, and 7 (1.0%) were either associate directors or directors of nurses.

### Perceptions of the Sharing Nurse Program

Close to half of the participants (300/694, 43.2%) were not aware of the Sharing Nurse program. Considering the nurses who knew about the program, only 6 had actually delivered services through a “Sharing Nurse” app. According to their answers, the factors that kept nurses from being a shared nurse included concern about personal safety (128/688, 18.6%), unclear regulations (126/688, 18.4%), lack of time (289/688, 42%), and dearth of energy (275/688, 40%). The concerns of the nurses are presented in Table 1.

**Table 1:** Concerns about home visiting as a shared nurse (N=694).

Concerns	Strongly unconcerned, n (%)	Partially unconcerned, n (%)	Neither concerned nor unconcerned, n (%)	Partially concerned, n (%)	Strongly concerned, n (%)	Missing, n (%)
Personal safety	16 (2.3)	9 (1.3)	57 (8.2)	82 (11.8)	522 (75.2)	8 (1.2)
Information security	26 (3.7)	38 (5.5)	97 (14)	143 (20.6)	383 (55.2)	7 (1)
Service quality supervision	18 (2.6)	25 (3.6)	120 (17.3)	164 (23.6)	357 (51.4)	10 (1.4)
Public recognition about shared nurse	33 (4.8)	35 (5.0)	128 (18.4)	145 (20.9)	345 (49.7)	8 (1.2)
Division of liability in medical disputes	11 (1.6)	8 (1.2)	41 (5.9)	59 (8.5)	568 (81.8)	7 (1.0)
Medical waste management	56 (8.1)	63 (9.1)	147 (21.2)	120 (17.3)	301 (43.4)	7 (1.0)
Service quality at home	18 (2.6)	25 (3.6)	120 (17.3)	164 (23.6)	357 (51.4)	10 (1.4)

Regarding the services that the participants think a shared nurse can deliver at home, the top three choices were blood collection for laboratory tests (489/694, 70.6%), intramuscular injection (473/694, 68.1%), and health education (508/694, 73.3%). The majority of the participants thought that at least 5 years of experience in bedside practice and the title of senior nurse were necessary qualifications to be a shared nurse.

Participants stated that they would consider being a shared nurse if they could obtain customized insurance (611/694, 88%), program training (488/694, 70.3%), clear statements on shared nurses’ rights and duties (580/694, 83.6%), real time position tracking services while delivering service (567/694, 81.7%), and alarm equipment (590/694, 85.0%). A few nurses also regarded video recording of the service procedure as essential. Table 2 presents the full details of these answers.

**Table 2:** Needs to meet to consider to be a shared nurse of participants (N=694).

Needs	Strongly disagree, n (%)	Partially disagree, n (%)	Neither agree nor disagree, n (%)	Partially agree, n (%)	Strongly agree, n (%)	Missing, n (%)
Permits from the hospital	12 (1.7)	9 (1.3)	50 (7.2)	49 (7.1)	562 (81.0)	12 (1.7)
Insurance	12 (1.7)	4 (0.6)	22 (3.2)	34 (4.9)	611 (88.0)	11 (1.6)
Video recording of the service procedure	30 (4.3)	30 (4.3)	90 (13.0)	104 (15.0)	427 (61.5)	13 (1.9)
Clear statements about shared nurses’ rights and duties	9 (1.3)	6 (0.9)	28 (4.0)	58 (8.4)	580 (83.6)	13 (1.9)
Real time position tracking while delivering service	14 (2.0)	8 (1.2)	37 (5.3)	57 (8.2)	567 (81.7)	11 (1.6)
Alarm equipment	10 (1.4)	3 (0.4)	26 (3.7)	54 (7.8)	590 (85.0)	11 (1.6)
Program training	11 (1.6)	12 (1.7)	69 (9.9)	101 (14.6)	488 (70.3)	13 (1.9)

More than half of the 694 participating nurses regarded the program as beneficial in terms of increasing public access to nursing care (n=483, 69.6%), extra income for nurses (n=402, 57.9%), preventing nurse turnover (n=371, 53.3%), and saving on health care costs (n=375, 54.0%). More than half (n=416, 60.0%) of the participants were optimistic about the program and were willing to be a shared nurse if there were clear and thorough regulations and supervision (Table 3).

**Table 3:** Perceived benefits toward sharing nurse program in China of participants (n=694).

Benefits	Strongly disagree, n (%)	Partially disagree, n (%)	Neither agree nor disagree, n (%)	Partially agree, n (%)	Strongly agree, n (%)	Missing, n (%)
More access to nursing care	40 (5.8)	29 (4.2)	129 (18.6)	140 (20.2)	343 (49.4)	13 (1.9)
Extra source of income for nurse	58 (8.4)	45 (6.5)	175 (25.2)	145 (20.9)	257 (37.0)	14 (2.0)
Preventing nurse turnover	82 (11.8)	66 (9.5)	160 (23.1)	119 (17.1)	252 (36.2)	15 (2.2)
Saving health costs for patients	66 (9.5)	57 (8.2)	182 (26.2)	129 (18.6)	246 (35.4)	14 (2.0)

### Variations in Perceptions of the Sharing Nurse Program

It was seen that older nurses (χ^2^_3_=32.926, *P*<.001), nurses with higher levels of education (χ^2^_3_=28.835, *P*<.001), and nurses from tertiary hospitals were more likely to be aware of the program. Male nurses were more willing to be shared nurses than female nurses (*Z*=–2.275, *P*=.02; see Tables 4 and 5).

**Table 4:** Nurses who were aware of the sharing nurse platform.

Groups			Participants, n/N (%)		Chi-square (*df*)		*P* value		Post hoc
**Age (years)**				32.926 (3)		<.001		C>B, B>A	
	A <25	47/120 (39.2)							
	B 25-34	235/423 (55.6)							
	C 35-44	81/108 (75.0)							
	D ≥45	30/43 (69.8)							
**Gender**				3.913 (1)		.048		N/A^a^	
	Male	17/22 (77.3)							
	Female	376/672 (56.0)							
**Education background**				28.835 (3)		<.001		D>C, C>B, D>A	
	A Diploma degree	8/22 (36.4)							
	B Associate’s degree	93/210 (45.1)							
	C Bachelor’s degree	271/449 (63.1)							
	D Master’s degree or above	13/13 (100.0)							
**Levels of working hospital**				18.669 (3)		<.001		A>B, A>D	
	A Tertiary hospital	205/313 (65.5)							
	B Secondary hospital	87/181 (48.1)							
	C Community health center	42/81 (51.9)							
	D Private hospital	57/114 (50.0)							

^a^N/A: not applicable.

**Table 5:** Attitude toward the perspective of the sharing nursing program.

Groups			Likert scale^a^, median (IQR)	Kruskal-Wallis test	*Z* score	*P* value
**Age (years)**				0.008	N/A^b^	>.99
	<25	2 (2-3)				
	25-34	2 (2-3)				
	35-44	2 (2-3)				
	≥45	2 (2-3)				
**Gender**				N/A	–0.140	.89
	Male	2 (2-3.25)				
	Female	2 (2-3)				
**Education background**				0.824	N/A	.84
	Diploma degree	3 (1-3)				
	Associate degree	2 (2-3)				
	Bachelor degree	2 (2-3)				
	Master degree or above	2 (2-3)				
**Levels of working hospital**				0.095	N/A	.99
	Tertiary hospital	2 (2-3)				
	Secondary hospital	2 (2-3)				
	Community health center	2 (2-3)				
	Private hospital	2 (2-3)				

^a^The item was rated on a 5-point Likert scale (1: extremely optimistic, 2: optimistic, 3: neither optimistic nor pessimistic, 4: pessimistic, 5: extremely pessimistic).

^b^N/A: not applicable.

## Discussion

To the best of our knowledge, this is the first study conducted to assess nurses’ perceptions of the Sharing Nurse program in China. Nurses were specifically targeted because they are the executive body of the Sharing Nurse program; therefore, their perceptions and insights are of vital importance to policy makers and for the effective implementation of the program.

We found that almost half (300/694, 43.2%) of the participants did not know about the ongoing Sharing Nurse program. This may be because the survey was conducted less than 1 month after the program was officially piloted, and therefore, there was limited time for the nurses to learn about it. It may also be because the official announcement had not been disseminated widely and efficiently, or the employer hospitals or community health care centers had not made attempts to take part in the program.

Home visiting should be a part of community-based family support services [1]. In fact, the health care system in China emphasizes the central role of community health care nurses in primary care and home visiting services [25,26]. However, the results showed that nurses from the community health centers were less likely to be aware of the Sharing Nurse program. Cogdill [27] found that nurses with a master’s degree were more perceptive to information needs more frequently than their colleagues. The low rate of community health care nurses who were aware of the Sharing Nurse program indicates, on the one hand, that community nurses, most of whom hold a diploma or associate’s degree, lack information. On the other hand, this also reflects a flaw in policy promotion among community health care centers, which should be the frontier of the Sharing Nurse program implementation.

The main reasons for the respondents not willing to be a shared nurse were work stresses, as 40% (275/688) of nurses reported they did not have time or energy after work. This is consistent with previous study findings in England and Australia, where nurses have reported that administrative work not related to the practical task of delivering home visits was a significant barrier to home visiting work [28]. In the study city, shared nurses affiliated to approved medical institutions have to sacrifice their own time to deliver home visiting services through Sharing Nurse platforms, which adds more stress to their already high level of workload [29]. China also faces a shortage of nurses, and the growth in the number of health care professionals does not meet the increasing demand [30]. The Sharing Nurse program is expected to reduce this imbalance in a more efficient way. Therefore, the number of nurses should be expanded to attract more individuals to the nursing profession and better motivate nurses to engage in the program.

Safety issues are also a major concern about the program that the respondents shared. Studies in other countries have found that clients who received home visiting services without prior knowledge were inclined to report concerns about their safety because they did not seek help but had been identified by others as potential beneficiaries of home visiting programs [31,32]. This can result in clients feeling vulnerable and powerless when they allow service providers into their homes [33]. Although we did not find any literature on clients’ safety concerns related to the Sharing Nurse program, we found that more than two-thirds of nurses were concerned about their personal safety. Thus, the safety of visiting homes is a concern not only for the clients but also for the nurses. Hence, to better implement the Sharing Nurse program, efforts should be made to ensure the nurses’ personal safety. There are many suggested measures to enhance home visiting safety. For example, the Sharing Nurse platforms verify the accuracy of information the clients upload, such as location, service requirements, and identity. The government may carry out strict inspections of the platforms. Necessary protective equipment such as real time positioning and alarm equipment could be provided to the shared nurses while delivering the home visiting service.

Liability division, when medical disputes occur while delivering a home visiting service through a Sharing Nurse platform, was found to be another major concern for the participating nurses. This concern relates to problems such as defining and dealing with medical disputes related to the services at home and whether accidents that happen to nurses when they are on the way to or back from the clients’ home are workplace accidents or not. This demonstrates that there is a need for clear regulations and practical guidelines. This is supported by the finding that over 80% (580/694) of the respondents agreed that “clear statements about shared nurses’ rights and duties” is necessary to the consideration of being a shared nurse. Previous findings also showed that the absence of related policies and practical guidelines was one of the major organizational barriers for Korean nurses to implement home visiting services [34].

Our study found that insurance is regarded by nurses to be essential to improve the program. Home visiting nurses face unique and complex risks in their workplaces as well as uncertainties on their way to and from clients’ homes. These nurses need insurance support including traffic accident insurance and personal accident insurance. Previous studies have also pointed out that a lack of professional indemnity insurance is an obstacle both for the employment of nurses and for the fulfilling of their professional role [35]. Additionally, during a labor shortage, employment-based benefits such as insurance can be used to help recruit and retain nurses [36]. Therefore, we recommend the formulation of customized insurance options for shared nurses by policy makers and relevant agencies.

Well-planned and effective preservice and ongoing training contributes to home visitor readiness and prepares them for the demands of the job [37]. According to our participants, program training is also needed. We, therefore, suggest tailoring nursing education and training to include aspects of home visiting for the benefit of prospective home visiting nurses.

Additionally, more than half of the respondents agreed that the program could be beneficial in saving on health care costs for patients, providing extra income for nurses, and preventing nurse turnover. Considering patients’ possible health care savings, the result is consistent with previous findings that home visiting could save costs such as parking fees, travel time, and time off work associated with travelling to a clinic [38]. It also cancels out the waiting time in hospital lines [24]. Shared nurses on the sharing nurse apps generally earn ¥100-300 (US $14-42) per visit for their services, which is more than what they could earn for their daily work [39]. Evidence [40,41] has shown that higher wages are associated with less turnover. The extra income, more flexibility, and the opportunities that the Sharing Nurse program offers make it more attractive to join the program.

The limitations of this study cannot be ignored. First, the participants in this study all came from one city in Shandong Province, which is not an official piloting area of the Sharing Nurse program. Nevertheless, Jinan is one of the earliest cities where the platform originated. Second, the convenience sampling method limits the generalizability of our findings. However, the sample of this study was purposefully selected to cover various levels of health care that are typical to China to explore differing perceptions among clinical nurses from different settings. Third, we only explored the perceptions of the Sharing Nurse program from nurses’ perspective, not from clients’ perspective. Future studies focusing on clients’ perceptions and the actual effectiveness of the program are warranted.

China’s Sharing Nurse program is a new way to facilitate home visiting services. However, in the early stage of the program, the awareness of the nurse program was low. The major concerns of the program include unclear liability division when medical disputes occur and potential personal safety issues. The participating nurses identified several needs that must be met for them to become a shared nurse, including insurance, permits from their employers, clear instructions concerning rights and duties, real time positioning while delivering the service, and one-key alarm equipment. Although the participating nurses noted many barriers of becoming a shared nurse, most of them held an optimistic view of the program and expressed their willingness to be part of it once regulations and practical guidelines are refined. The findings contribute to promoting knowledge about China’s Sharing Nurse program and, hopefully, provide implications for further refinement and regulations of the program.

## Abbreviations

e-questionnaires: electronic questionnaires
